# Metastasis of spine from adenoid cystic carcinoma of the parotid gland: two case reports 

**DOI:** 10.1186/s13256-023-03926-w

**Published:** 2023-05-15

**Authors:** Saeid Safaei, Parisa Azimi, Taravat Yazdanian

**Affiliations:** 1 Knee and Sport Medicine Research Center, Milad General Hospital, Tehran, Iran; 2 Neuroscience Research Center, Shahid Beheshti University of Medical Sciences, Arabi Ave, Daneshjoo Blvd, Velenjak, Tehran, 19839-63113 Iran; 3 School of Medicine, Capital Medical University, Beijing, China

**Keywords:** Adenoid cystic carcinoma, Parotid gland carcinoma, Spinal metastasis

## Abstract

**Background:**

Spinal metastasis from adenoid cystic carcinoma of the salivary gland is extremely rare. We present two interesting cases of spinal metastasis from adenoid cystic carcinoma of the parotid gland.

**Case summary:**

A 29-year-old Persian male and a 48-year-old Persian female presented with parotid gland mass. The two patients received parotidectomy and radiotherapy. The pathological examination result was adenoid cystic carcinoma. Because of intractable back pain, patients were referred to the hospital after 7 years and 9 months, respectively. Both cases underwent spinal surgery. Histopathology confirmed spinal metastasis from adenoid cystic carcinoma of the parotid gland (case 1: T6, T12, and L1; case 2: T12). Anterior corpectomy of T12 and lateral screw fixation at T11 and L1 were done in case 2. Posterior spinal fusions from T2 to L3 and from T10 to L2 were performed in case 1 and case 2, respectively. Both patients showed good clinical improvement. The last follow-up (case 1: 24 months; case 2: 6 months after surgery), plain radiographs and computed tomography scan showed good fusion without instrumental failure and magnetic resonance imaging revealed good decompression of the spinal cord of both cases.

**Conclusion:**

Although spinal metastasis from adenoid cystic carcinoma of the parotid gland is extremely rare, it is necessary to be careful in the differential diagnosis.

## Introduction

Adenoid cystic carcinoma (ACC) is an infrequent malignant neoplasm of the major salivary glands [1]. The majority of salivary gland tumors occur in the parotid glands. ACCs account for about 1% of all malignancies of the head and neck area [1, 2]. The tumor is usually slow growing compared with other carcinomas, and the frequent sites of metastasis are lungs, bone, liver, and, rarely, the brain [3]. Spinal metastasis from ACC of the salivary glands is an exceedingly rare situation, and only a few case reports exist in the literature [3–5]. Here, we present two rare cases of spinal metastasis from ACC of the parotid gland. The clinical summary, imaging findings, and surgical procedures are discussed.

## Case presentation

### Case 1

**Fig. 1:**
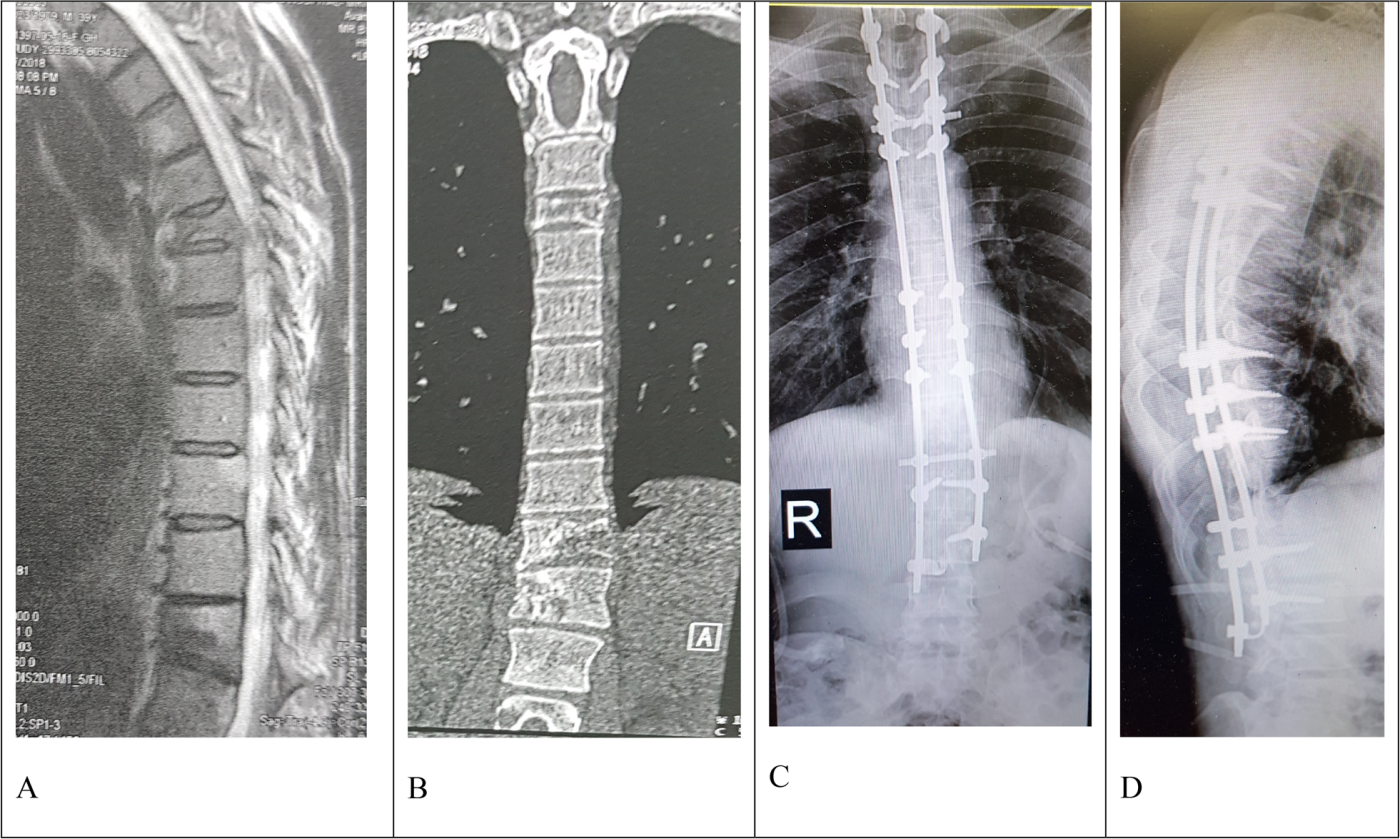
Spinal metastases from parotid adenoid cystic carcinoma in case 1. **a**, **b** Preoperative sagittal T2-weighted magnetic resonance imaging and coronal computerized tomography scan revealed multilevel significant bone destruction and pathological vertebral fractures. **c**, **d** Postoperative posteroanterior and lateral X-ray films

A 29-year-old Persian man with a left parotid gland mass was referred for ultrasonography of the parotid region, which indicated a well-defined irregular hypoechoic lesion in the deep and superficial lobes of the left parotid gland. One week later, he had a parotidectomy of the left parotid gland with ipsilateral selective lymph node dissection. The pathological examination result was ACC. The treatment was followed by parotid area and neck radiotherapy with a total dose of 50 Gy in 2011. Afterward, he was frequently followed-up for 5 years. Seven years after primary surgery, the patient was referred to a spine surgeon because of back pain and kyphosis. On clinical examination, he had normal neurological status. Computed tomography (CT) scan and magnetic resonance imaging (MRI) evaluation showed T6, T12, and L1 vertebral metastasis (Fig. 1a, b). Posterior spinal fusion from T2 to L3 was performed. The postoperative pathology established a metastatic ACC of the spine. The 2 year postoperative follow-up examination and radiological imaging showed that the patient has normal neurological status and normal sagittal alignment (Fig. 1c, d).

### Case 2

**Fig. 2:**
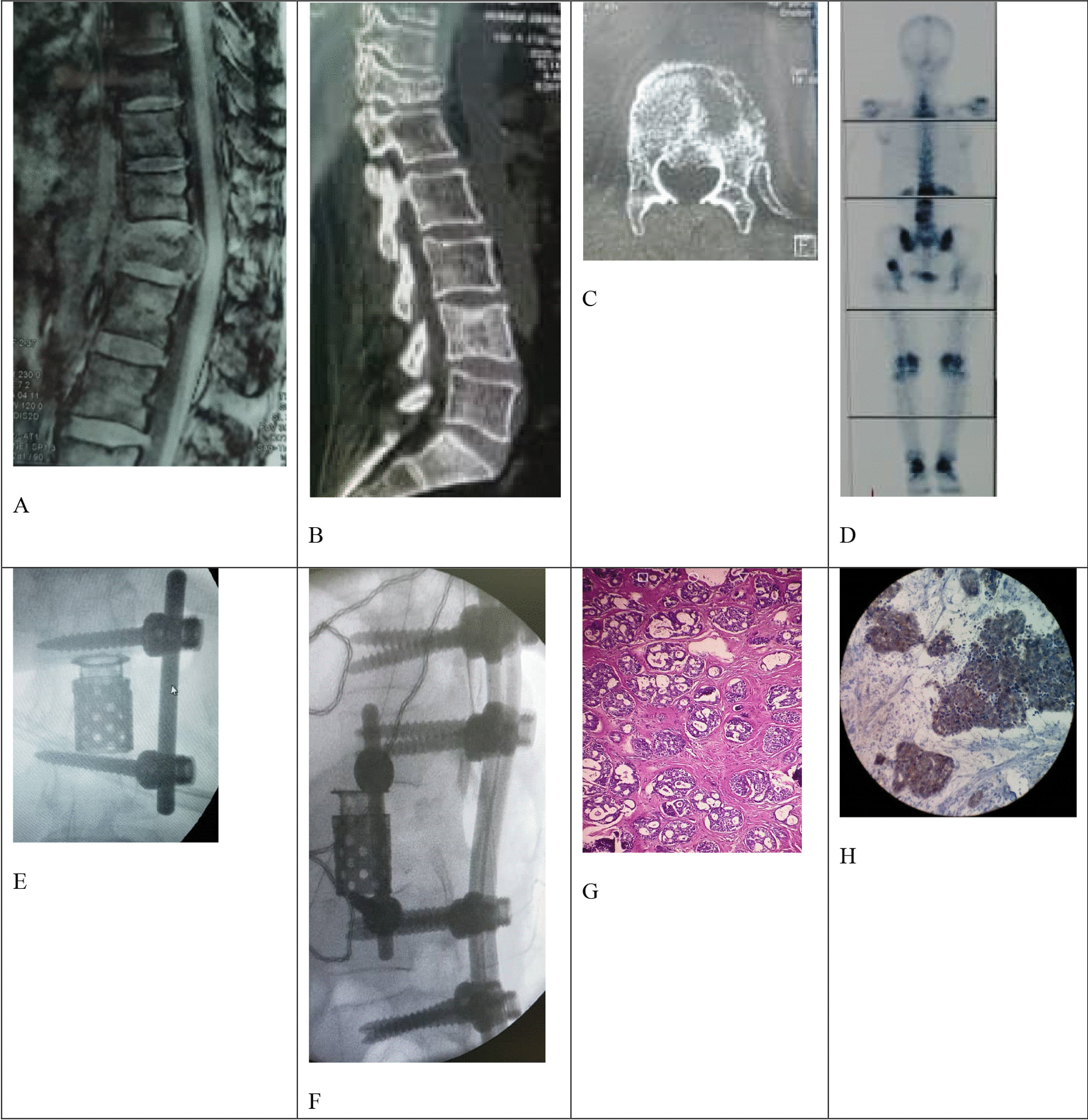
Spinal metastases from parotid adenoid cystic carcinoma in case 2. **a**–**c** Preoperative sagittal magnetic resonance imaging, sagittal and axial computerized tomography scan showed bone destruction, pathological vertebral fracture, and cord compression. **d** The whole-body bone scan indicated increased uptake in T12 and left acetabulum due to bone metastasis. **e**, **f** Intraoperative Anterior Posterior and lateral C-arm X-ray imaging. **g**, **h** Histopathology confirmed metastatic adenoid cystic carcinoma from a parotid gland of the spine

A 48-year-old Persian woman was referred to the clinic with a right parotid mass. Head and neck MRI showed a lobulated tumoral mass with heterogeneous enhancement in the right parotid gland without lymph node involvement. Fine needle aspiration (FNA) reported malignancy changes. Two weeks later, the patient received the right total parotidectomy. The pathological report indicated ACC in the excised mass, and the surgical margin was free from the tumor. Lymph vascular invasion was not indicated. The treatment was followed by radiotherapy to the parotid area and the neck (total dose 54 Gy). Afterward, she was regularly followed-up. Nine months after primary surgery, the patient was evaluated with spinal MRI because of back pain. She had T12 and L4 vertebral metastasis and received spinal radiotherapy (total dose 25 Gy). After 2 months, she presented with progressive back pain and was admitted to the Milad Hospital, Tehran, Iran, in March 2020. MRI, CT, and bone scan showed a T12 significant bone destruction, pathological vertebral fracture, and spinal cord compression (Fig. 2a–d). There was no neurological deficit. Treatment by anterior corpectomy of T12 and lateral screw fixation at T11 and L1 was performed. After 2 days, posterior spinal fusion from T10 to L2 was done (Fig. 2e, f). Both spinal surgeries were performed under intraoperative neuromonitoring. Histopathology confirmed spinal metastasis from ACC of the parotid gland (Fig. 2g, h). The case had no postoperative complications. She left the hospital on the fifth postoperative day in a good health state. Six months after the surgery, she has regularly been followed-up and has no neurological problems. Patient follow-up will continue for at least 2 years.

## Discussion

In these two case reports, we have presented our experience with spinal surgery in the management of two patients who had spinal metastasis from ACC of the parotid gland. Spinal instrumentation surgery can improve the survival and overall quality of life (QoL) of patients with spinal metastasis, but a correct diagnosis is essential to delivering effective treatment. Multimodality adjuvant therapy will be helpful to improve the prognosis of such cases.

To the best of our knowledge, only one case of spinal metastasis that was reported as the first clinical sign of parotid ACC has been presented in the literature [4]. Their patient (a 68-year-old female), like our two patients, was successfully managed by surgical procedure but radiation therapy was not performed [4]. Researchers have suggested the posterior approach for spinal decompression when the spinal metastases have caused neurological symptoms, especially myelopathy and radiculopathy [4]. They recommended radiation therapy in incomplete excision cases or residual disease [4]. Due to microscopic involvement in the resected margins, we recommend radiation therapy after spine surgery even with grossly resected lesions.

MRI and/or CT should be applied to confirm suspected spinal metastasis. Myelography, single-photon emission computed tomography (SPECT), and positron emission tomography (PET) may be used in the surveillance of patients with suspected spinal metastasis [6]. If tissue for pathologic diagnosis is necessary and there is no primary metastasis, a patient could benefit from a biopsy [6]. In our two cases, the imaging modality, such as MRI, CT, and bone scan, was applied to establish a diagnosis of spinal metastasis, and vertebral biopsy was not used. Although there is no consensus on the management of the metastatic disease, complete illness control might be attained for early detection of spinal metastasis and more optimal treatment with new technology such as PET, PET/CT, and precision medicine. These technologies were not applied in this report; however, it is recommended to use new technologies, especially in developed countries.

Spinal bone metastases cause pathologic fractures, leading to back pain and nerve damage. These fractures represent significant pathology that reduces a patient’s quality of life, hence early diagnosis and suitable treatment are mandatory. Accessible treatments can be invasive or noninvasive such as chemotherapy, or radiotherapy. There is insufficient evidence for a treatment protocol to be suggested in patients with spinal metastases. The management of these patients needs a multidisciplinary team of experts including oncologists and spine surgeons. The Neurologic, Oncologic, Mechanical, Systemic factors (NOMS) framework helps to make treatment decisions for cases with spinal metastatic tumors. The neurologic component includes the assessment of the grade of epidural spinal cord compression and neurological status of the patient, the oncologic component explains the radio-sensitivity of primary cancer, the mechanical evaluation of the spine determines the requirement for spinal column stabilization, and the systemic component considers the general systemic condition of the patient [7]. In these cases, the NOMS algorithm was used to decide on surgery treatments and complementary treatments were considered.

## Conclusions

Spinal metastasis may be suspected in patients with ACC of the parotid gland with signs and symptoms not explained by the primary site. Instrumentation surgery should be performed in cases with parotid ACC who develop spinal metastasis, based on the NOMS framework.

## Data Availability

Not applicable.
